# Trends in the use of personal protective equipment by health care workers who experienced occupational accidents in Brazil

**DOI:** 10.47626/1679-4435-2020-567

**Published:** 2021-02-11

**Authors:** Leonel Lucas Smith de Mesquita, Arlene de Jesus Mendes Caldas, Vanessa Moreira da Silva Soeiro, Sâmea Cristina Santos Gomes, Thais Furtado Ferreira

**Affiliations:** 1 Departamento de Enfermagem, Universidade Federal do Maranhão (UFMA) - São Luís (MA), Brazil; 2 Departamento de Saúde Pública, UFMA - São Luís (MA), Brazil

**Keywords:** personal protective equipment, health care workers, occupational accidents

## Abstract

**Introduction:**

Personal protective equipment creates a protective barrier for mucous membranes, airways, and skin in situations with possible exposure to biological material.

**Objectives:**

To analyze temporal trends in the use of personal protective equipment at the time of accidental exposure to biological materials in Brazilian health care workers.

**Methods:**

This was an ecological time series study of the use of personal protective equipment in health professionals who experienced accidental exposure to biological materials. Data were analyzed by region and federal unit, as well as in the country as a whole. Temporal trends were investigated using Prais-Winsten regression models with calendar year as the independent variable.

**Results:**

The use of personal protective equipment increased by 4.62% at a national level. Increases were also observed in all regions of the country. Temporal trends within federal units, however, showed significant heterogeneity.

**Conclusions:**

Though the use of personal protective equipment increased in the country as a whole, the rate of change varied widely between federal units. These findings highlight the need for changes in public policy and the implementation of permanent education programs for health care professionals, especially in regions with lower or stable rates of personal protective equipment use.

## INTRODUCTION

According to the World Health Organization (WHO), 3 million health care workers are exposed to blood-borne pathogens every year as a result of percutaneous exposure incidents.^1^ In Brazil, the Information System for Health Notifications (Sistema de Informação de Agravos de Notificação; SINAN) received 48,105 reports of incidents involving biological material in 2013, and 46,951 such reports in 2014.^2^ Given the risks associated with exposure to biological materials, the Centers for Disease Control and Prevention (CDC) in the United States has published a set of safety instructions for health care professionals known as the Standard Precautions.^3^ These are to be implemented during patient care and when handling contaminated material, regardless of confirmed or suspected patient infection, by employees of all types of health facilities.^4^ The standard precautions aim to minimize the risk of exposure to biological materials through measures such as hand hygiene, sharps safety practices, and use of personal protective equipment (PPE) such as gloves, gowns, face masks, boots, protective eyewear and face shields.^5^ PPE creates a protective barrier for mucous membranes, airways, and skin in situations with possible exposure to biological material.^6^

In Brazil, ordinance No. 3.214/78 establishes work health and safety regulations, implementing protective actions and providing legal guarantees of health and safety for workers in their occupational environment. Regulatory norm 6 (NR6)^7^ refers specifically to the use of PPE, which should be mandatory for all workers and offered at no cost by their employers, while regulatory norm 32 (NR32) addresses the health and safety of health care workers, setting basic guidelines for the implementation of protection and safety measures for these individuals.^8,^9^,10^

Studies on the use of PPE have yielded concerning results. In an analysis of nationwide data, Miranda^2^ calculated prevalence rates of 68.1%, 38.5% and 22.2% for glove, gown and mask use, respectively; the corresponding figures in the state of Bahia were 69.5%, 36.5% and 30.7%, according to Cordeiro.^11^ Studies by Almeida,^12^ in Manaus, and Vieira,^13^ in Florianópolis, used a different methodological approach to reveal similarly concerning findings, noting that 71% of health care workers had been wearing at least one type of PPE when at the time of the incident. In the study by Correa et al.,^14^ in the state of Maranhão, the percentage of individuals wearing three or more types of PPE during a possible exposure incident was 41.3%. Most studies in the literature have examined this issue in specific geographical locations, such as a single city or hospital, or even a single hospital sector.^12,13,15^ The few nationwide studies on PPE and exposure to biological material have only analyzed this issue using descriptive methods.^11,^14^^

Nationwide studies on PPE use^2,16^ are scarce, and have never investigated this topic using time series analysis. In light of these observations, this study adopted a time series approach to examine nationwide data on the subject, so as to contribute to decision-making and public policy planning for health care workers.^17^ The time series method may be especially helpful in the context of this study as it could reveal the effects of initiatives to encourage the use of PPE and improve incident reporting. Therefore, the aim of this study was to perform a time series analysis of the prevalence of PPE use at the time of accidental exposure to biological materials (AEBM) in Brazilian health care workers.

## METHOD

This was an ecological time series study of PPE use by health professionals who experienced AEBM. Data were analyzed at 3 levels: country (Brazil), region (north, northeast, midwest, southeast and south) and federal unit (26 states and the federal district). The study population consisted of all health professionals who experienced AEBM from January 1, 2010 to December 31, 2016. AEBM was defined as any incident involving blood or other biological fluids, experienced by health care professionals during occupational activities with exposure to potentially infectious materials.^18^

A database was constructed using data on AEBM extracted from the SINAN records, available from the Informatics Department of the Unitary Health System (Departamento de Informática do Sistema Único de Saúde; DATASUS) on the website of the Integrated Environmental and Worker Health Program (Programa Integrado em Saúde Ambiental e do Trabalhador; PISAT) of the Collective Health Institute of the Universidade Federal da Bahia (ISC/UFBA). The following variables were extracted for each incident: federal unit of occurrence (26 states or federal district); occupation of the affected worker (health care professionals: doctors, dentists, nurses, nutritionists, pharmacists, psychologists, physical therapists, nursing technicians and assistants, dental technicians and laboratory technicians) according to the Brazilian Classification of Occupations; year of occurrence (2010 to 2016); and PPE (gloves, gowns, face mask, boots, protective eye wear and face shields).

Descriptive methods were used to calculate frequencies and percentages of occurrence of AEBM in health care professionals. The prevalence of PPE use in these cases was then calculated. The presence of three or more types of PPE was considered sufficient protection against biological risks, since the standard precautions state that those at risk of coming into contact with any bodily fluids, regardless of suspected infection, must use four types of PPE (gloves, gown, protective eye wear, face mask).^19,20^ The proportion of incidents where PPE was worn was calculated for each year, federal unit, region and for the country as a whole. This was done by dividing the number of incidents where PPE was worn by the total number of incidents and multiplying the result by 100, in the following manner:

### PROPORTION = NUMBER OF INCIDENTS WHERE PPE WAS WORN/TOTAL NUMBER OF INCIDENTS X 100

Temporal trends in PPE use during occupational accidents were examined using Prais-Winsten^21^ regression models with calendar year as the independent variable. Regression parameters were estimated using a first‐order autocorrelation correction. This technique is superior to simple linear regression in that it controls for serial autocorrelation in the time series. The results were then examined to verify whether PPE use followed an upward, downward or stable trend. This was done by analyzing regression coefficients, with positive values suggesting an increase and negative values showing a decrease. Rates with nonsignificant regression coefficients (p > 0.05) were considered stable. Data were analyzed using STATA software, version 14.0.

In accordance with the requirements of National Health Council (Conselho Nacional de Saúde) resolution No. 466/2012,^22^ this study was evaluated and approved by the Research Ethics Committee of the Hospital Universitário Presidente Dutra at the Universidade Federal do Maranhão (HUUFMA), under protocol No. 2.039.925/2017.

## RESULTS

**Figure 1:**
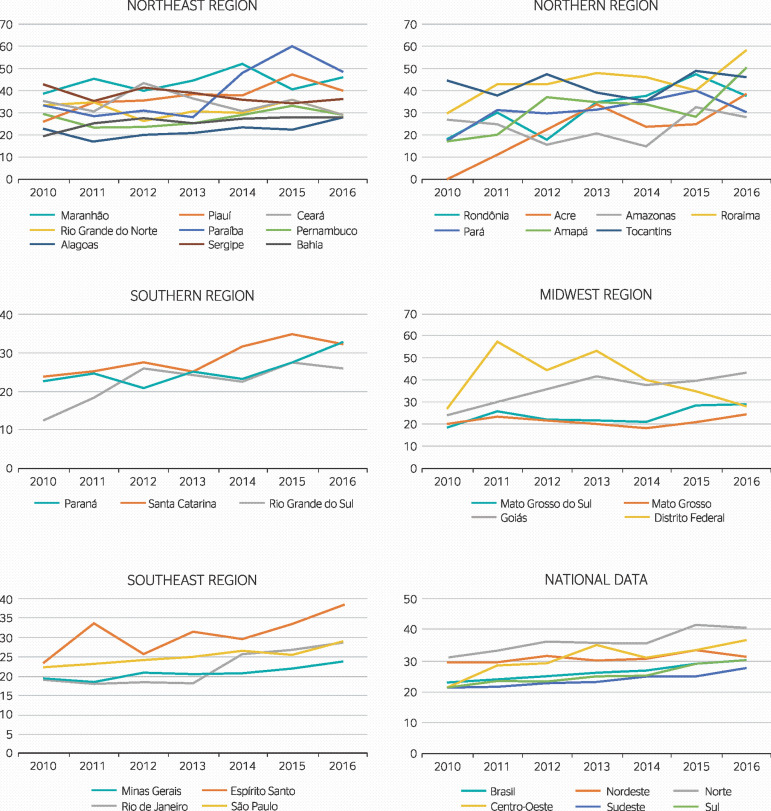
Frequency of personal protective equipment use by health care workers at the time of accidental exposure to biological materials in Brazil, per region and federal unit, from 2010 to 2016.

A total of 331,603 incidents of AEBM were recorded in the SINAN from 2010 to 2016. Health care workers were involved in 243,621 (73.42%) of these cases. Only 37,464 (15.38%) individuals wore 3 or more types of PPE at the time of the exposure incident, corresponding to a mean of 5,352 incidents per year. The highest nationwide rate of PPE use was observed in 2016 (30.25%) and the lowest, in 2010 (22.96%). Regional rates were highest in northern Brazil in 2015 (41.60%) and lowest in southern Brazil in 2010 (21.25%) (Figure 1).

The time series analysis showed that PPE use increased by 4.62% at a national level during the study period. Increases were also observed across all regions of the country, though the rate of change varied significantly between federal units (Table 1).

**Table 1:** Temporal trends in the use of personal protective equipment by health care workers at the time of accidental exposure to biological materials in Brazil, per region and federal unit, from 2010 to 2016.

Variables	Coefficient	p-value*	Trend	Rate of change
				(%)
Brazil	0.019647	0.000	Upward	4.62
Region				
North	0.0187593	0.001	Upward	4.41
Rondônia	0.0632734	0.002	Upward	15.68
Acre	0.0753197	0.075	Stable	-
Amazonas	0.0102488	0.697	Stable	-
Roraima	0.0259235	0.046	Upward	6.15
Pará	0.0359949	0.037	Upward	8.64
Amapá	0.0583086	0.031	Upward	14.36
Tocantins	0.0061458	0.493	Stable	-
Northeast	0.006737	0.009	Upward	1.56
Maranhão	0.0071909	0.257	Stable	-
Piauí	0.0305327	0.001	Upward	7.28
Ceará	-0.008026	0.402	Stable	-
Rio Grande do Norte	-0.0016229	0.849	Stable	-
Paraíba	0.0473792	0.040	Upward	11.52
Pernambuco	0.0131756	0.264	Stable	-
Alagoas	0.023746	0.018	Upward	5.61
Sergipe	-0.0107386	0.043	Downward	-2.44
Bahia	0.0202271	0.041	Upward	4.76
Midwest	0.0292092	0.010	Upward	6.95
Mato Grosso do Sul	0.0225047	0.069	Stable	-
Mato Grosso	0.0075644	0.493	Stable	-
Goiás	0.0398465	0.014	Upward	9.60
Federal District	-0.0178722	0.452	Stable	-
Southeast	0.0175894	0.000	Upward	4.13
Minas Gerais	0.0146717	0.001	Upward	3.43
Espírito Santo	0.0192342	0.009	Upward	4.52
Rio de Janeiro	0.0356263	0.010	Upward	8.54
São Paulo	0.0147109	0.000	Upward	3.44
South	0.0240172	0.000	Upward	5.68
Paraná	0.0200914	0.044	Upward	4.73
Santa Catarina	0.0281815	0.002	Upward	6.70
Rio Grande do Sul	0.0467877	0.039	Upward	11.37

* p-value calculated using the Prais-Winsten time series method.

Source: MS/CGSAT/SINAN (2017).^23^

The North region showed an upward trend in PPE use (4.41%), though the rate of change varied across federal units. Trends were stable in the states of Acre, Amazonas and Tocantins, but increased in Rondônia, Roraima, Pará and Amapá. The largest changes in the country were observed in the states of Rondônia (15.68%) and Amapá (14.36%) (Table 1). The Northeast region also showed an increase in PPE use over time (1.56%), but its rate of change was the lowest across all regions examined. Variations between federal units were more significant, though the state of Sergipe was the only one in which PPE use decreased over time. Trends were stable in the states of Maranhão, Ceará, Rio Grande do Norte and Pernambuco, but increased in Piauí, Paraíba, Alagoas and Bahia (Table 1). The Midwestern region also showed an increase in PPE use over time (6.95%), though the analysis of federal units in the region revealed that only the state of Goiás showed a similarly increasing trend. The rate of PPE use in the states of Mato Grosso, Mato Grosso do Sul and Distrito Federal were stable throughout the study period (Table 1). The south (5.68%) and southeast (4.13%) regions showed increasing temporal trends, and were the only ones in which all federal units trended in the same direction, with the largest change observed in the state of Rio Grande do Sul (11.37%) (Table 1).

## DISCUSSION

The present study revealed a growing trend in the use of PPE during incidents involving AEBM throughout Brazil and its geographical regions. This finding may be the result of public policy on occupational accidents and PPE. The obligation to report occupational accidents was imposed by regulation 777/2004^24^ and reiterated in regulation 104/2011.^25^ Mandatory weekly reports were instituted by ordinance 1271/2014.^26^ NR32,^10^ published in 2005 by the Ministry of Work and Employment, aimed to protect and promote the health and safety of health care workers in occupational settings. In 2010, the need to conduct oversight of this regulation resulted in the addition of Annex III, which established a committee for the prevention of occupational accidents involving biological materials in health care services.^27^ Yet the introduction of policies on occupational accidents and PPE is a relatively recent development in Brazil, dating just prior to the time period analyzed in the present study, which may explain the low notification rate and lack of safety at the start of the study period, as well as the improvement observed over time. Nevertheless, the rate of improvement on these measures still appears to be quite small.

The results obtained when data were analyzed by federal unit were even more concerning; though all southern and southeastern states showed improvements over time, the findings from other regions were far more variable. The rate of PPE use remained stable in many northern, northeastern and midwestern states, while in some cases, such as the state of Sergipe, it actually decreased over time. These variations may be explained by the technical, social, cultural and political characteristics of each federal unit, all of which may lead to individual differences in PPE use and incident reporting among workers.^28,29^ According to Souza,^16^ the northeastern region of Brazil has the highest rate of missing data in incident report forms, while the southeast region has the lowest.

In absolute numbers, the southern and southeastern regions reported the most incidents in the study period, while the northern and northeastern regions reported the fewest. Interestingly, these findings suggest that the regions with the most complete records and the highest number of incident reports are the ones that show upward trends in PPE use, while the trends observed in regions with more missing data and fewer reports show greater variability.

Economic and territorial issues must also be considered when interpreting these findings. Larger states with more fragile economies may have more difficulty implementing measures to decrease the risk of accidents. Initiatives such as the provision of safety equipment, better working conditions, PPE and safety training may not be possible in under-resourced regions.^29^ This may be the case of the northern and northeastern regions of Brazil.

Strengths of the present study include its comprehensive nature, which provides a national profile of the topic of study rather than focusing on a single city of hospital, as well as the implications of its findings. The low rates of PPE use during incidents with AEBM and the temporal trends of this metric at a national, regional and statewide level identify several regions in need of more intense intervention. The methodological approach used is also a strength of this study. Time series analysis is a highly effective method of estimating temporal changes in data and making prognostic predictions. Additionally, no other studies in the national or international literature have applied this approach to the issue of PPE use.

One limitation of the present study was the presence of underreporting, an issue often faced in secondary data analysis, even in the presence of a mandatory reporting system.^31^ This may influence the assessment of the topic of study. However, the presence of underreporting does not reduce the relevance of the present findings. A second limitation is the methodological variability of previous studies of this issue. Regional and nationwide studies on the topic are scarce, both in the national and international literature, with the few existing studies limited to descriptive approaches rather than in-depth analysis.

## CONCLUSION

The use of PPE during AEBM has increased in Brazil and its subregions. Upward trends in this metric were observed in all southern and southeastern states, though growth rates were still small. Slight upward trends were also noted in other regions in the country, though some showed no growth at all during the study period. These results may contribute to strategies to improve public policy and support the development of new actions and initiatives to reduce the occurrence of occupational accidents in health care settings. The present findings also highlight the need for permanent education programs for health care professionals, especially in regions with lower and stable rates of PPE use.
